# Gold nanoarray deposited using alternating current for emission rate-manipulating nanoantenna

**DOI:** 10.1186/1556-276X-8-295

**Published:** 2013-06-24

**Authors:** Jiancai Xue, Qiangzhong Zhu, Jiaming Liu, Yinyin Li, Zhang-Kai Zhou, Zhaoyong Lin, Jiahao Yan, Juntao Li, Xue-Hua Wang

**Affiliations:** 1 State Key Laboratory of Optoelectronic Materials and Technologies, School of Physics and Engineering, Sun Yat-Sen (Zhongshan) University, Guangzhou 510275, People’s Republic of China; 2 State Key Laboratory for Biocontrol, School of Life Science, Sun Yat-Sen (Zhongshan) University, Guangzhou 510275, People’s Republic of China

**Keywords:** Anodic aluminum oxide template, Au nanoarray, Emission rate, Nanoantenna, Surface plasmon, 82.45.Yz, 78.47.jd, 62.23.Pq

## Abstract

We have proposed an easy and controllable method to prepare highly ordered Au nanoarray by pulse alternating current deposition in anodic aluminum oxide template. Using the ultraviolet–visible-near-infrared region spectrophotometer, finite difference time domain, and Green function method, we experimentally and theoretically investigated the surface plasmon resonance, electric field distribution, and local density of states enhancement of the uniform Au nanoarray system. The time-resolved photoluminescence spectra of quantum dots show that the emission rate increased from 0.0429 to 0.5 ns^−1^ (10.7 times larger) by the existence of the Au nanoarray. Our findings not only suggest a convenient method for ordered nanoarray growth but also prove the utilization of the Au nanoarray for light emission-manipulating antennas, which can help build various functional plasmonic nanodevices.

## Background

Excited by an incident photon beam and provoking a collective oscillation of free electron gas, plasmonic materials gain the ability to manipulate electromagnetic field at a deep-subwavelength scale, making them play a major role in current nanoscience [1-5]. The plasmonic metallic nanostructures have presented a vast number of potential applications in various prospective regions such as plasmon lasers [6-8], optical tweezers [9,10], and biochemical sensing platforms [11-13]. In particular, the nanoarray structure attracts extensive research efforts for its strong coupling between adjacent nanorods, which leads to dramatic field enhancement and high energy transfer efficiency [14,15], resulting in a diverse range of plasmonic devices such as subwavelength color imaging, plasmonic waveguide, functional metamaterials [15-19], as well as nanoantennas for manipulating the light emission of various nanoemitters, which is of great importance for quantum science, nano-optical communication, and light-harvesting devices [20-25].

Owing to the self-organized hexagonal arrays of uniform parallel nanochannels, anodic aluminum oxide (AAO) film has been widely used as the template for nanoarray growth [26-29]. Many distinctive discoveries have been made in the nanosystems fabricated in AAO films [30-34]. As increasing emphasis is placed on low cost, high throughput, and ease of production, AAO template-assisted nanoarray synthesis is becoming the method of choice for the fabrication of nanoarrays [35].

However, due to the existence of a barrier layer, it is impossible to grow nanoarrays instantly after the AAO template has been prepared via a two-step anodization process using direct current (DC). Some complicated processes must be included, such as the Al foil removing, the barrier layer etching, and the conducting layer making. The pregrowth processes dramatically increase the difficulty of AAO template-assisted nanoarray synthesis especially in the case that a thin AAO film with a few micrometer is required [18]. On the other hand, it is reported that alternating current (AC) can get across the barrier layer and implement direct metal array deposition [36-38]. However, using the AC method, it is difficult to grow the nanoarray as ordered as that using DC, which leads to poor field enhancement and broad surface plasmon resonance (SPR) peaks [18,36-38]. This flaw prevents the AC growth method from being widely used.

In this paper, we propose a pulse AC metal nanoarray growth method, which can cut off some inevitable complicated processes in AAO DC deposition and easily fabricate metallic nanoarrays as uniform as those by DC deposition. The extinction spectra, the quantum dot (QD) emission rate manipulation measurement, as well as the theoretical analysis of electric field distribution and local density of states (LDOS) confirm that the pulse AC-grown Au nanoarrays can be a good candidate for nanoantennas.

## Methods

### Preparation of samples

The AAO templates were prepared by a two-step anodization process [18,33]. First, the aluminum sheets (purity 99.999%) were degreased in acetone and electropolished under a constant current condition of 1.2 A for 3 min in a mixture of HClO_4_ and C_2_H_5_OH at 0°C to smooth the surface morphology. In the first and second anodization processes, treated aluminum sheets were exposed to 0.3 M H_2_SO_4_ or H_2_C_2_O_4_ solution under a constant voltage of 19 or 45 V in an electrochemical cell at a temperature of about 4°C. The alumina layer produced by the first anodization process was removed by wet chemical etching in a mixture of phosphoric acid (0.15 M) and chromic acid (0.60 M) at 60°C for 1 h. The barrier layer of AAO templates was thinned stepwise with reducing potential down to 6 V.

The ordered Au nanoarrays were deposited in the nanopores of the AAO template by pulse AC (50 Hz) electrodeposition in an electrolyte containing HAuCl_4_ (10 mM) and H_2_SO_4_ acid (0.03 M) with a Pt counter electrode. The deposition was carried on instantly after the completion of the AAO template using a common AC power source (GW APS-9301, GW Instek, New Taipei City, Taiwan) supplying a 4-s pulse of 16 V, followed by a growth potential of 9 V. There is no need to remove the Al foil, etch the barrier layer, and make a conducting layer before Au nanoarray growth, which makes the electrodeposition very convenient. The normal AC deposition method was carried on in the same condition as the pulse AC, except for the 4-s pulse of 16 V.

The quantum dots were commercial carboxyl CdSe/ZnS quantum dots, which were purchased from Invitrogen Corporation (Carlsbad, CA, USA). In the time-resolved photoluminescence (PL) measurement of the QDs, the Al foil was taken using CuCl_2_ solution, and QDs were dropped on the barrier side of the AAO template.

### Characterization of samples

Scanning electron microscopy (SEM) was performed using a Zeiss Auriga-39-34 (Oberkochen, Germany) operated at an accelerating voltage of 5.0 kV. Transmission electron microscopy (TEM) was performed using a JEOL 2010HT (Akishima-shi, Japan) operated at 100 kV. The TEM samples were prepared by dissolving the AAO template containing Au nanoarrays in NaOH solution. The extinction spectra were recorded using an ultraviolet–visible-near-infrared region (UV–vis-NIR) spectrophotometer (PerkinElmer Lambda950, Waltham, MA, USA) using a *p*-polarized source with an incident angle of 70°.

### Optical experiments

The PL from the samples was collected by the reflection measurement. An *s*-polarized laser for the measurements of PL was generated using a mode-locked Ti:sapphire laser (MaiTai, Spectra Physics, Newport Corporation, Irvine, CA, USA) with a pulse width of approximately 150 fs and a repetition rate of 79 MHz. The wavelength of the laser beam was tuned to 400 nm. The scattering noise was filtered using a band-pass filter, followed by a 100-mm-focal-length lens which was used to excite the sample at a Brewster angle *θ*_b_ ≈ 50°. The luminescence from the sample was collected using the focusing lens and a long-wave pass filter before entering the liquid-nitrogen-cooled CCD (SPEC-10, Princeton Instruments, Trenton, NJ, USA). The time-resolved PL decay traces were recorded using a time-correlated single-photon counting system (PicoQuant GmbH, Berlin, Germany).

### Computational simulations

The computational simulations were performed using the finite difference time domain (FDTD) method with Bloch and perfectly matched layer (PML) boundary conditions for the *x-* and *y-*axes and *z*-axis, respectively. The cell size was 2 × 2 × 5 nm^3^. The nanowires were hexagonally arrayed with a rod diameter of 34 nm, length of 150 nm, and spacing of 110 nm. The refractive index of Al_2_O_3_ was set to be 1.76, and the complex dielectric constants of the gold were taken from the literature of Johnson and Christy [39].

The photonic LDOS was obtained by calculating the Green function with the help of the COMSOL software (version 4.2a). The hexagonal lattice of Au nanowires was simulated with the scale of 7 × 7 arrays. The lattice constant was set to be 110 nm. The length and the diameter of each Au nanowire were set to be 150 and 34 nm, respectively. The refractive index of the background was 1.76. The dielectric constant of gold was taken from the literature of Johnson and Christy [39]. An electric point dipole is set 10 nm above the center of the arrays. A block with the size of 0.99 × 0.887 × 0.31 μm^3^ is set to separate the array and the PML. The PML is set to a size of 1.65 × 1.547 × 1.15 μm^3^ with general type. To get a good mesh, a sphere with the radius of 4 nm is set to surround the dipole. The mesh inside the block is predefined as fine. The mesh of the PML is predefined as extra fine to get good absorption. The scattering boundary is set to the outside of the PML.

## Results and discussion

**Figure 1:**
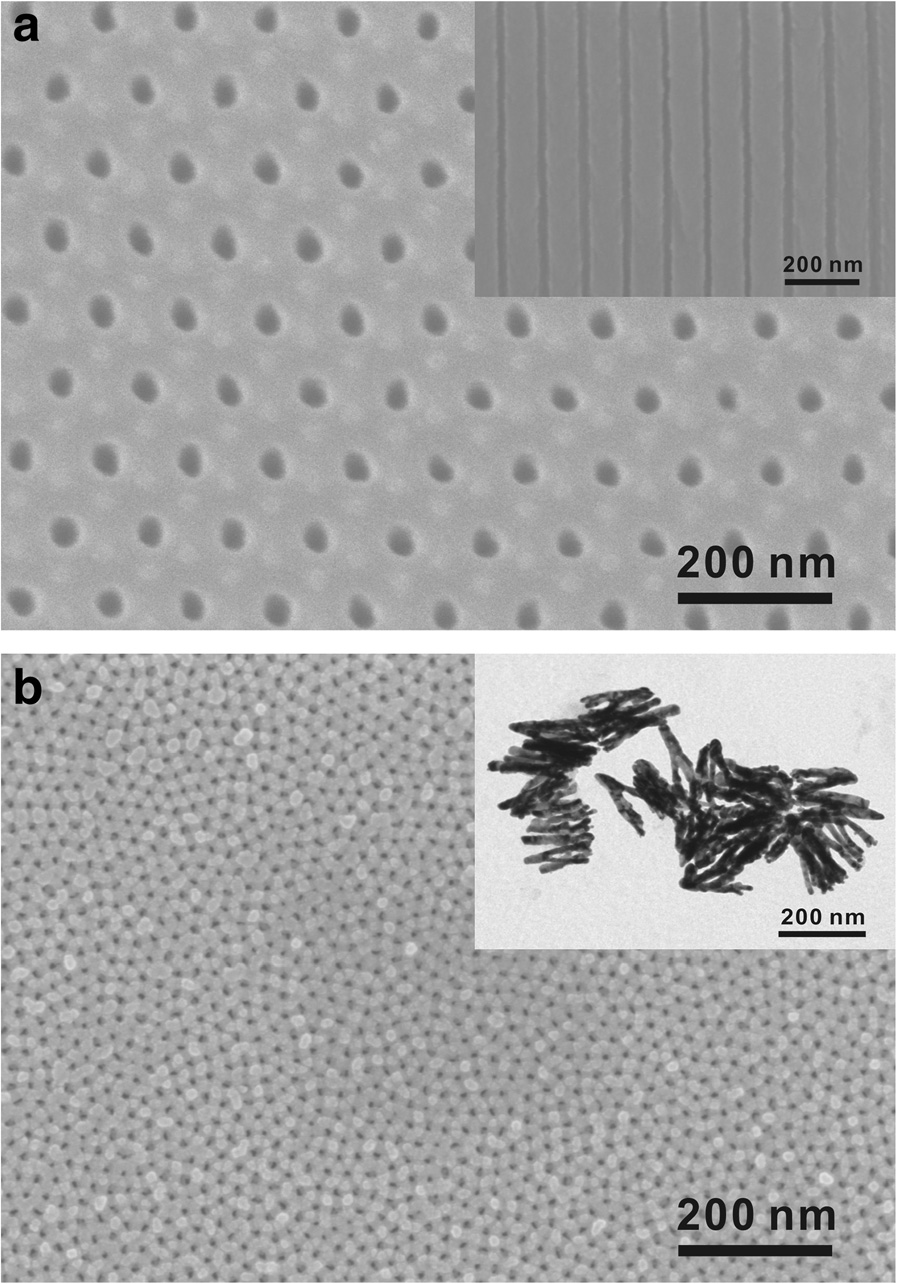
**SEM and TEM characterization of samples.** (**a**, **b**) The top SEM view of AAO templates with uniform hexagonal nanochannels prepared using H_2_C_2_O_4_ and H_2_SO_4_, respectively. The estimated average diameter *d* and period *a* are *d* = 34 nm and *a* = 110 nm (**a**) and *d* = 20 nm and *a* = 50 nm (**b**). The inset of (**a**) is the cross-sectional SEM view of the AAO template made in H_2_C_2_O_4_, and the inset of (**b**) is the TEM image of AC-grown Au nanowires in the AAO template manufactured by H_2_C_2_O_4_ anodization, with the average diameter and length being 34 and 150 nm, respectively.

Figure 1 shows the SEM and TEM images of the sample characterization. Figure 1a,b shows the top SEM views of AAO templates with uniform hexagonal nanochannels prepared using H_2_C_2_O_4_ and H_2_SO_4_, respectively. The estimated average diameter *d* and period *a* of the AAO template prepared using H_2_C_2_O_4_ are *d* = 34 nm and *a* = 110 nm, and those of the AAO template anodized in H_2_SO_4_ are *d* = 20 nm and *a* = 50 nm.

The inset of Figure 1a is the cross-sectional SEM view of the AAO template made in H_2_C_2_O_4_. It can be seen that the nanochannels are very vertical, which makes it possible to grow highly ordered nanoarrays. The TEM image of Au nanowires is presented in the inset of Figure 1b. These Au nanowires were grown in the AAO template manufactured by H_2_C_2_O_4_ anodization, with the average diameter and length being 34 and 150 nm, respectively. It should be noted that the Au nanowires in the inset TEM image were deposited by the pulse AC method, which made the highly ordered growth possible. On the other hand, the good length uniformity as well as high occupied rate can hardly be achieved using the normal AC method (see Additional file 1: Figures S1 and S2).

**Figure 2:**
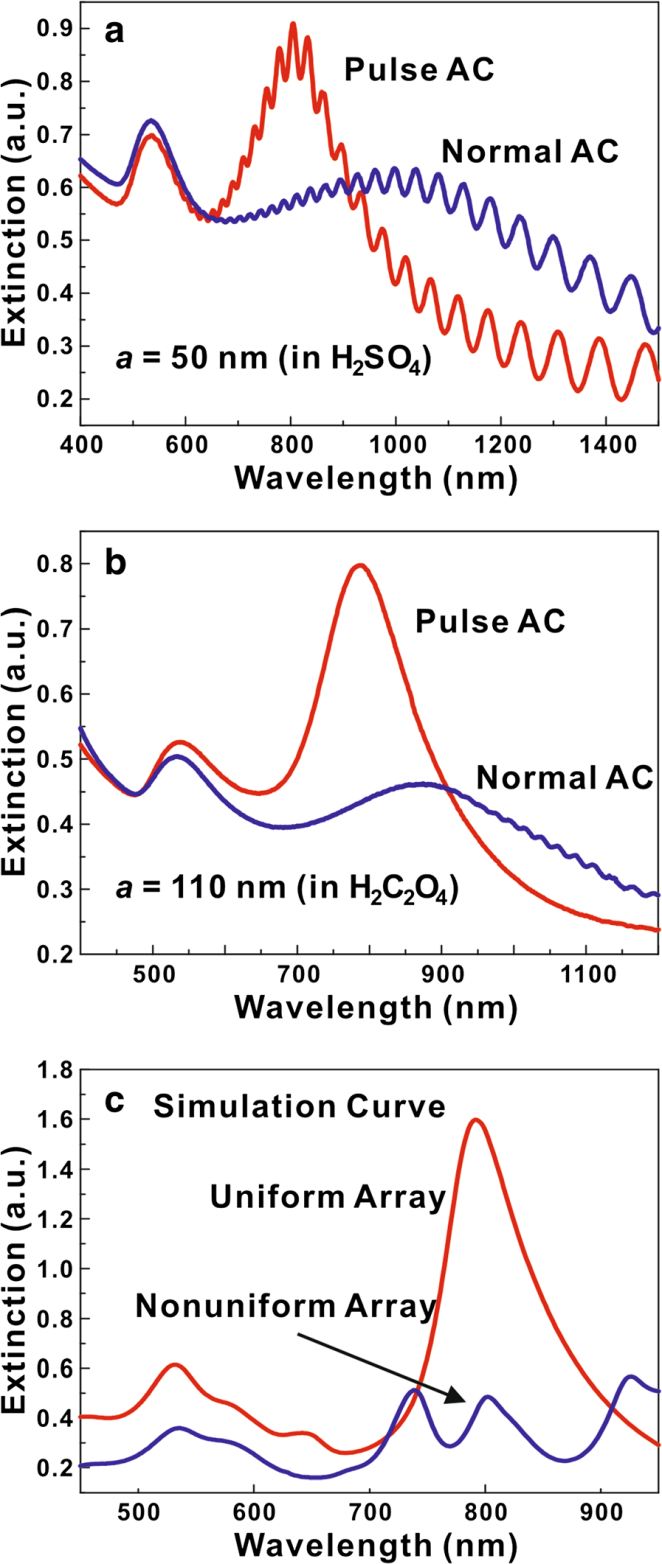
**Experimental and simulation extinction spectra of Au nanoarrays prepared by pulse AC and normal AC methods.** (**a**, **b**) Experimental extinction spectra of the Au nanoarrays grown in AAO prepared using H_2_SO_4_ and H_2_C_2_O_4_, respectively. (**c**) Simulation extinction spectra of the uniform and nonuniform Au nanoarrays with period *a* = 110 nm and diameter *d* = 34 nm. The length of the uniform nanoarray is set to be 150 nm. The simulation unit cell of the nonuniform nanoarray contains six nanowires with the length *L* = 50, 75, 100, 125, 150, and 200 nm.

Figure 2 is the extinction spectra of the Au nanoarrays prepared by pulse AC and normal AC methods. The extinction value is defined as −ln(*T*/*T*_0_), where *T* is the transmittance of the Au nanoarray and *T*_0_ is the reference transmittance, according to previous reports [40-42]. Figure 2a,b shows the experimental results of Au nanoarrays, grown in the AAO template with period *a* = 50 and 110 nm, respectively. The oscillations in Figure 2a are due to the Fabry-Pérot resonance of the AAO template, and this result is similar to our previous work [33]. The red curves represent samples deposited by the pulse AC method, while the blue curves represent the Au nanoarray made by normal AC deposition. Using a *p*-polarized source with an incident angle of 70°, two peaks appear at the extinction spectra, which can be attributed to the transverse and longitudinal surface plasmon resonances (abbreviated by TSPRs and LSPRs, respectively), caused by free electrons near the metal surface oscillating perpendicularly to and along the long axis of the nanoarrays [40,41]. The extinction intensity ratio of LSPRs to TSPRs in the Au nanoarray deposited by pulse AC is much larger than that in the normal AC-prepared Au nanoarray, and the full width at half maximum (FWHM) of the extinction peak is much narrower. It should be noted that the extinction curve of pulse AC-grown Au nanoarray is quite similar to that of DC-grown Au nanoarray in many remarkable works [14,40-42], and this is a strong demonstration of the high growth quality of our method. Although the pulse method has been reported in DC deposition by Nielsch et al. before [43], the pulse AC method is seldom reported in previous works.

To further discuss the extinction spectra results, we used the FDTD method to calculate the extinction spectra of uniform and nonuniform nanoarrays (Figure 2c). The length of a single nanowire in the uniform Au nanoarray is set to be 150 nm according to TEM images, and the basic simulation unit cell of the nonuniform Au array contains six nanowires with the length *L* = 50, 75, 100, 125, 150, and 200 nm (simulation model, see Additional file 1: Figure S3). From Figure 2c, it is obviously seen that the extinction intensity ratio of LSPRs to TSPRs decreases dramatically in the nonuniform nanoarray structure (blue curve), and this phenomenon fits quite well with the experimental result. There are several LSPR peaks appearing at the nonuniform nanoarray extinction spectra, which are caused by the LSPRs of Au nanowires with different length. For the normal AC-grown nanoarrays, the length of a single Au nanowire varies continuatively, so numerous LSPR peaks can finally broaden the FWHM of the Au nanoarray extinction spectra. Furthermore, it is interesting to note that the LSPR location of simulation data fits quite well with the experimental results (788 nm in experiment, 792 nm in simulation). Due to the strong SPRs in the pulse AC-grown Au nanoarray, it is believed that the uniform Au nanoarray can generate large enhancement of electric field and local density of states, which makes the Au nanoarray a good candidate for nanoantennas. Thus, we use the FDTD and Green function methods to do our further theoretical investigation.

**Figure 3:**
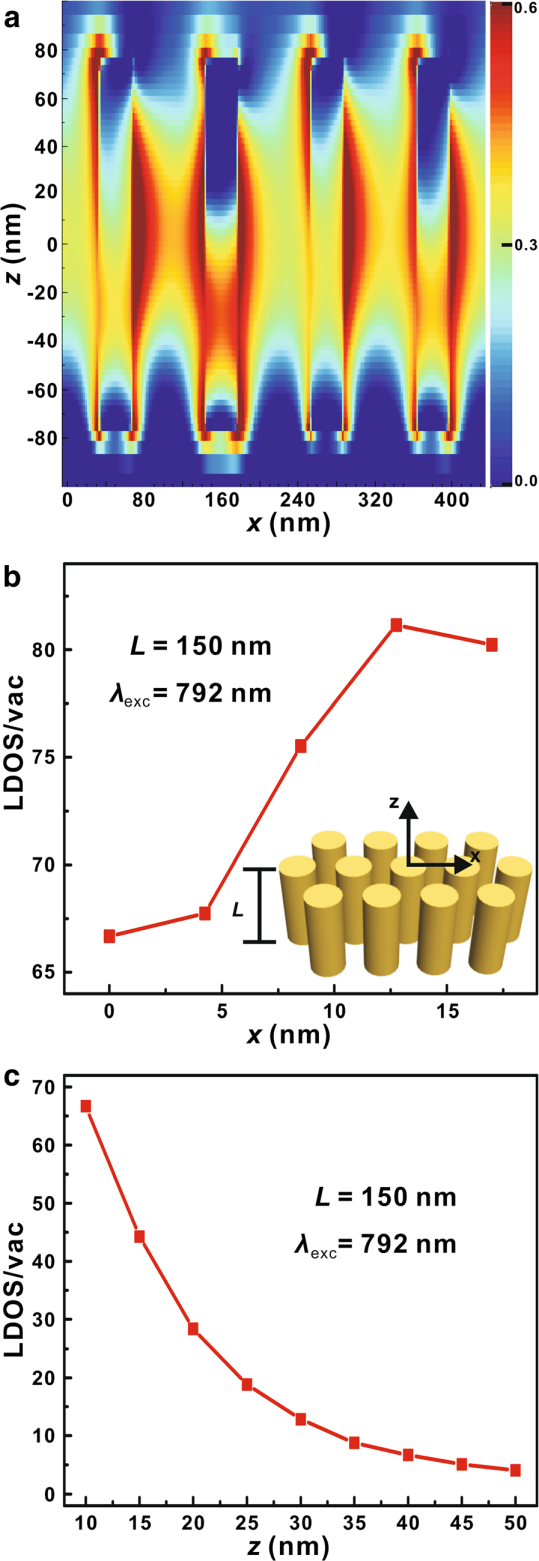
**Field distribution and LDOS enhancement.** (**a**) The field distribution of Au nanoarray (*L* = 150 nm, *d* = 34 nm, *a* = 110 nm) at the plane wave wavelength of 792 nm with an incident angle of 40°. (**b**) The *x*-position dependence of LDOS enhancement at the wavelength of 792 nm. As shown from the sketch of the simulation model in the inset, the zero point is at 10 nm above the center Au nanowire. The enhancement of LDOS at the center and the edge is 66.7 and 81.2, respectively. (**c**) The *z*-position dependence of LDOS enhancement.

Figure 3 shows the field distribution of the Au nanoarray with *L* = 150 nm, where the incident light is a plane wave at the wavelength of 792 nm with an incident angle of 40°. The field intensity enhancements are drawn at the logarithmic scale. The large field enhancement at every tip of the Au nanoarray is clearly seen, and this field enhancement can cause the increment of LDOS. However, the electric field tends to concentrate at some certain nanowire in the nonuniform Au nanoarray, and this asymmetric field distribution decreases the whole extinction intensity and displays nonuniform field enhancement which may affect the stability and repeatability of the Au nanoarray in the application of nanoantennas (see Additional file 1: Figure S3). Furthermore, with the help of the Green function, the LDOS is given as [44]:

ρr→,ω=2ωπc2ImtrGr→,r→,ω,

where Im stands for the imaginary part and tr denotes the trace of the Green tensor matrix in brackets.

From the Maxwell equations, one can get E→r→,ω=iωμ0∫dr→'G↔r→,r→',ω⋅j→r→,ω By setting a dipole source j→r→',ω=j→ωδr→'−r→0 the Green function can be calculated by the electric field at the position of the dipole as $\vec{E}\left({\vec{r}}_{0},\omega \right)=\mathrm{i\omega}\mu_{0}\overset{↔}{G}\left({\vec{r}}_{0},{\vec{r}}_{0},\omega \right)\cdot \vec{j}\left(\omega \right)$.

Also, the matrix form of $\vec{E}\left({\vec{r}}_{0},\omega \right)$ can be written as:

$$E=\mathrm{i\omega}\mu_{0}\left[\begin{array}{ccc} G_{\mathrm{xx}} & G_{\mathrm{xy}} & G_{\mathrm{xz}}\\ G_{\mathrm{yz}} & G_{\mathrm{yy}} & G_{\mathrm{yz}}\\ G_{\mathrm{zx}} & G_{\mathrm{zy}} & G_{\mathrm{zz}} \end{array}\right]\cdot \left[\begin{array}{c} j_{x}\\ j_{y}\\ j_{z} \end{array}\right].$$

After choosing three $\vec{j}$ of different directions, all the elements of the Green matrix can be obtained so as to get the LDOS.

The LDOS is calculated by the finite element method with the help of the COMSOL software (version 4.2a). As shown in Figure 3b, one can see that the LDOS enhancement at 792 nm is much larger at the edge which is in accord with the field distribution in Figure 3a, and the maximum enhancement is 81.2 times (define the LDOS enhancement as the ratio of LDOS around the nanoarray to LDOS in vacuum). The *z*-position dependence of LDOS enhancement shows that the LDOS enhancement decreases dramatically with the increase of distance at the *z*-axis, and the LDOS enhancement phenomenon nearly disappears at the point of 50 nm above (see Figure 3c). If we can control the *z*-distance between the nanoemitter and the Au nanoarray, it is possible to manipulate the LDOS enhancement as well as the light emission rate. Moreover, the large field and LDOS enhancement can also be demonstrated by the PL measurement [33,45], and these detailed experimental results can be found in Additional file 1: Figure S4.

**Figure 4:**
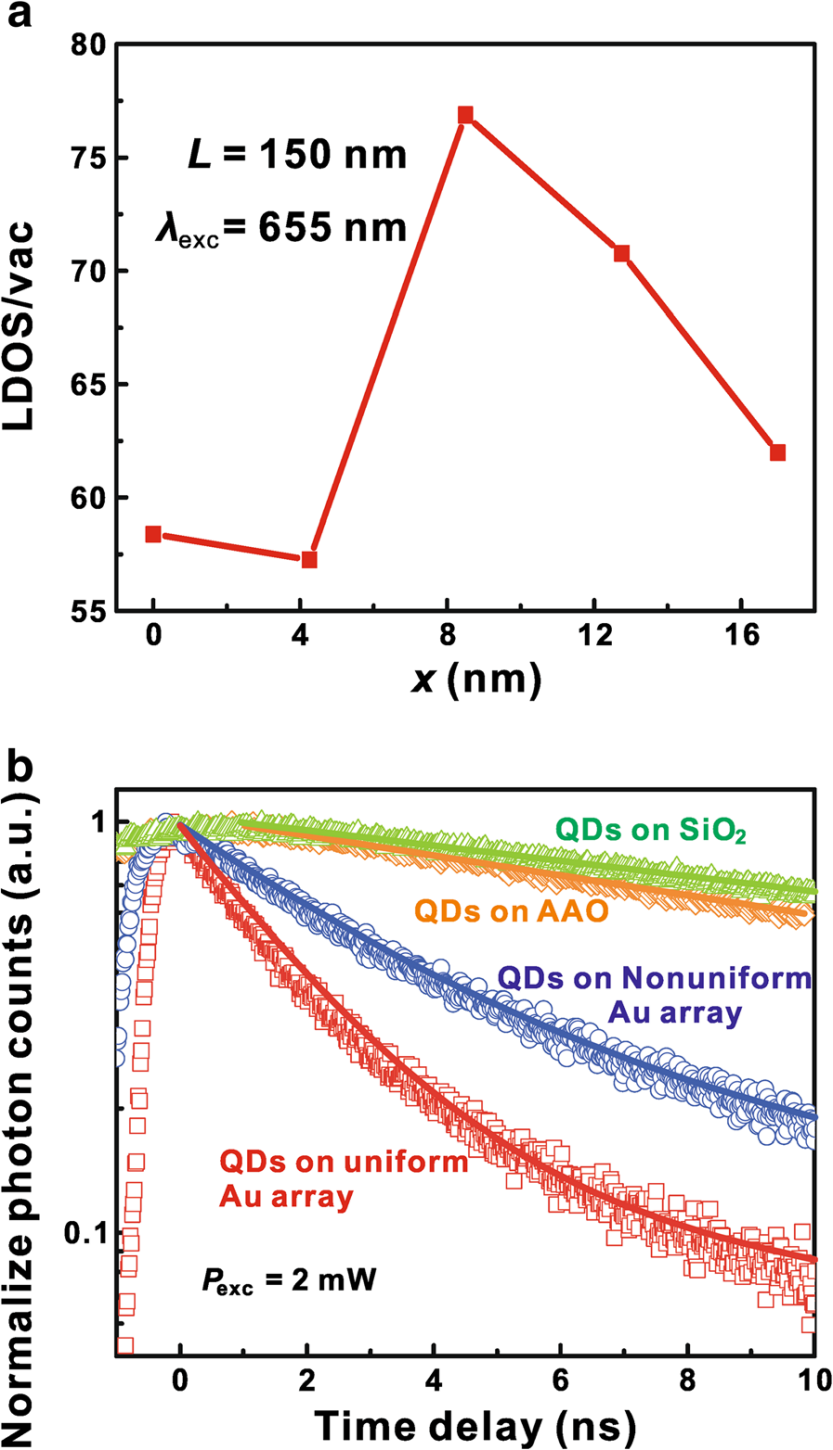
**LDOS enhancement and the normalized time-resolved PL spectra of QDs on Au nanoarray.** (**a**) The *x*-position dependence of LDOS enhancement at the wavelength of 655 nm. An average LDOS enhancement of 64 times can be achieved. (**b**) The normalized time-resolved PL spectra of QDs with emission peak located at 655 nm. The emission rate of QDs increases from 0.0429 to 0.5 ns^−1^ by the existence of the Au nanoarray, showing an enhancement of 10.7 times.

Since the emission rate of nanoemitters is proportional to the LDOS, the increase of LDOS greatly confirms the utilization of the Au nanoarray for light emission-manipulating nanoantennas. The light emission rate manipulation experiment was set up using a time-correlated single-photon counting system [45], and the normalized time-resolved PL spectra are shown in Figure 4. The nanoemitters were commercial quantum dots with emission peak located at 655 nm, and the wavelength of incident laser was tuned to 400 nm with the excitation power of 2 mW. Figure 4a shows the LDOS enhancement in the presence of a dipole with an emission wavelength of 655 nm at 10 nm above the Au nanoarray. An average enhancement of 64 times can be found from the calculation results. Compared with the average LDOS enhancement of 75 times at the emission wavelength of 792 nm, it can be seen that the LDOS enhancement region of the Au nanoarray is quite large, which can make the Au nanoarray find useful applications in the design of functional plasmonic devices. In Figure 4b, the PL decay trace of the QDs on SiO_2_ substrate and pure AAO are single exponential with the corresponding emission rate *τ* = 0.0429 ns^−1^ on SiO_2_ and *τ* = 0.0559 ns^−1^ on pure AAO. The single-exponential decay trace indicates that the cooperative effects caused by the assembling of QDs can be neglected [18]. On the contrary, the time-resolved PL curve of QDs on Au nanoarray decays in a two-component exponential form:

$$I_{\mathrm{PL}}\left(t\right)=A_{f}e^{-t/t_{f}}+A_{s}e^{-t/t_{s}},$$

where *A*_f_ and *A*_s_ are the weight factors of the fast and slow decay processes, respectively, and *t*_f_ and *t*_s_ are the corresponding lifetimes (emission rate *τ* = 1/*t*). The two-component exponential decay form suggests the strong interaction between QDs and Au nanoarrays.

From the data in Figure 4, *t*_s_ is 23.3 ns, while *t*_f_ is 2.0 and 3.4 ns for QDs on uniform and nonuniform Au nanoarrays, respectively. As the enhancement of LDOS decreases dramatically with the increase of distance (see Figure 3c), the slow decay process may indicate a part of the QDs which are quite far from the Au nanoarray, and the fast decay process can be attributed to the QDs that are located at the LDOS-enhancing region. From the fitting data, the emission rate of the QDs on the uniform Au nanoarray increased from 0.0429 to 0.50 ns^−1^, showing an enhancement of 10.7 times. As the distance between QDs and Au nanoarray is variable (QDs cannot assemble at the top side of the Au nanoarray) and the LDOS enhancement is sensitive to the increase of the *z* distance, it is reasonable that the light emission rate enhancement is smaller than the average theoretical LDOS enhancement. Also, it should be noted that the normalized *A*_f_ rate (*A*_f_ / (*A*_f_ + *A*_s_)) for QDs on uniform and nonuniform Au nanoarrays is 87.4% and 76.1%, which means that the fast decay process is dominant and the uniform Au nanoarray is a better choice for emission-manipulating nanoantennas. This Au nanoarray is the sample in Figure 2b, which is similar to the uniform simulation model of Figure 3, and the time-resolved PL spectra of QDs with emission peak located at 790 nm on the Au nanoarray can be found in Additional file 1: Figure S5.

## Conclusions

In this letter, we have proposed an easy and controllable method to prepare highly ordered Au nanoarrays by pulse alternating current deposition in anodic aluminum oxide template. This method not only averts some complicated inevitable processes in AAO DC deposition but also can easily fabricate Au nanoarrays as uniform as those by the DC deposition, which can be demonstrated using SEM image, TEM image, and UV–vis-NIR spectrophotometer. Using the FDTD and Green function methods, we further theoretically investigated the surface plasmon resonance, electric field distribution, and LDOS enhancement in the uniform Au nanoarray system and found that the maximum LDOS enhancement can be 81.2 times at the tip of the Au nanoarray. The time-resolved PL spectra of quantum dots show that the Au nanoarray can increase the emission rate of QDs from 0.0429 to 0.5 ns^−1^ (10.7 times larger). Our findings reveal that the conveniently AC-grown Au nanoarray can serve as light emission-manipulating antennas and could help build various functional plasmonic nanodevices.

## Abbreviations

AAO: Anodic aluminum oxide; AC: Alternating current; DC: Direct current; FDTD: Finite difference time domain; FWHM: Full width at half maximum; LDOS: Local density of states; PL: Photoluminescence; PML: Perfectly matched layers; QDs: Quantum dots; SEM: Scanning electron microscopy; SPR: Surface plasmon resonance.

## Competing interests

The authors declare that they have no competing interests.

## Authors’ contributions

JX, ZKZ, ZL, and JY prepared the samples. JX, QZ, and ZKZ anticipated the optical experiments and analyzed the related experiment data. JX, ZKZ, and YL characterized the morphology of the samples. JX and ZKZ performed the simulations using FDTD solution and interpreted the simulation results. JML, JTL, and XHW performed the numerical simulation of the LDOS section. ZKZ proposed the pulse AC growth method and finalized the manuscript. All authors read and approved the final manuscript.

## Supplementary Material

Additional file 1 **Supporting information.** The file contains Figures S1 to S5. Click here for file
