# Supplementary material for: Gold nanoarray deposited using alternating current for emission rate-manipulating nanoantenna
Source: Nanoscale Res Lett. 2013 Jun 24;8(1):295. doi: 10.1186/1556-276X-8-295 (PMC3694511; doi:10.1186/1556-276X-8-295)
Supplement: Additional file 1 — Supporting information. The file contains Figures S1 to S5. [file 1556-276X-8-295-S1.pdf]

Supporting Information for

" Gold Nanoarray Deposited by Alternative Current for Emission Rate Manipulating Nanoantenna ", *by* Jiancai Xue, Qiangzhong Zhu, Jiaming Liu, Yinyin Li, Zhang-Kai Zhou*, Zhaoyong Lin, Jiahao Yan, Juntao Li, and Xuehua Wang*

1. The TEM and cross section SEM images of Au nanoarrys grown by pulse AC and normal AC method

Figure S1a and b display the Au nanoarrys grown by pulse AC and normal AC method after the AAO template was dissolved by NaOH. It is obviously to see the nanowires in Figure S1a which were prepared by pulse AC method is quite uniform, while the length of normal AC growth Au nanaowire is nonuniform. Also it should be noted that the growth quality of pulse AC growth shown in Figure S1a is nearly the same with nanowires grown by DC method [1,2].

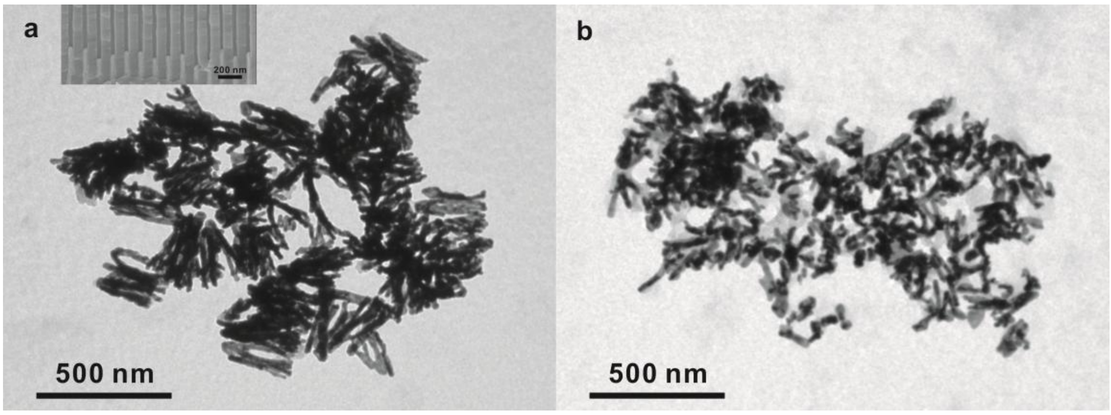

Figure S1. The TEM images of Au nanoarrys grown by pulse AC (a) and normal AC method (b). The inset image in Figure S1a is the typical cross section SEM image of Au nanoarray loaded in the AAO template.

The TEM image strongly suggests that the pulse method greatly improve the growth quality of AC deposition, and reaches the level of widely used DC method

(the TEM samples are from the extinction samples in Figure 2b). The inset image in Figure S1a is the typical cross section SEM image of Au nanoarray loaded in the AAO template, from which we could see the Au nanoarray is highly ordered.

2. The back SEM view of Au nanoarrays grown by pulse AC and normal AC method

The main reason that prevents normal AC deposition method to get good growth uniformity may be the poor occupied rate of the AAO nanochannel. Since there are many empty holes (Figure S2b), the deposition current would distribute unevenly, leading to the length nonuniform of the grown nanoarray. It is reported that using a relatively high voltage pulse at the beginning of nanoarray growth could much improve the occupied rate [3]. Although the pulse growth method was proposed in DC growth, it also works in AC deposition process. From the Figure S2a, one can clearly see the uniform Au nanoarray after the barrier layer was removed by H_3PO_4, and the occupied rate is nearly 100%.

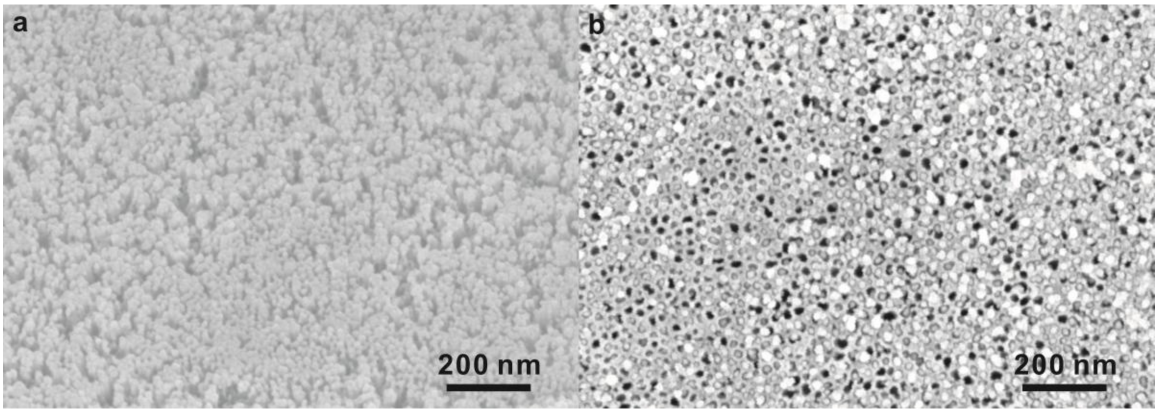

Figure S2. The back SEM view of Au nanoarrays grown by pulse AC (a) and normal AC (b) method.

3. The FDTD calculated field distribution of nonuniform Au nanoarray

From Figure 2c, we could see the extinction curve of nonuniform Au nanoarray displays 3 peaks at 738, 802, and 929 nm, which indicate the plasmon resonance mode of nanowires with different length, and the field distribution of these plasmon resonance modes is presented in Figure S3. Figure S3a is the simulation unit cell of the nouniform nanoarray, which contents 6 nanowires with the length *L* = 50, 75, 100, 125, 150 and 200 nm. As the field distribution is nearly the same, we showed the results of 4 nanowires in the Figure S3b-S3d. The field intensity enhancements are drawn at the logarithmic scale. .

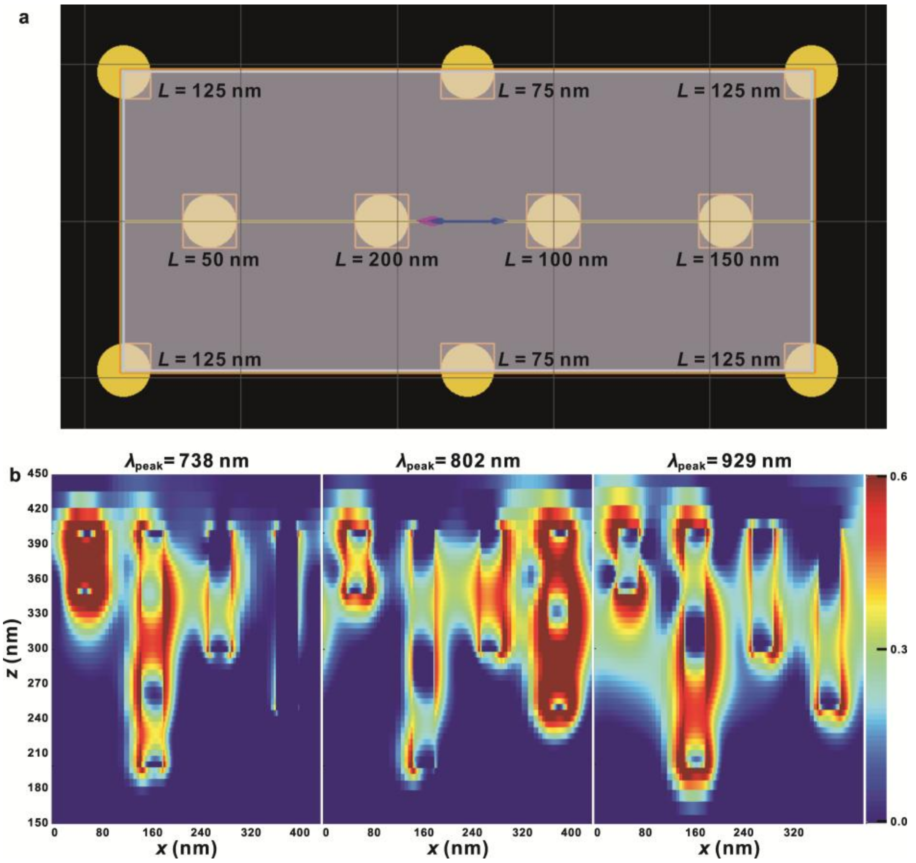

Figure S3. The FDTD calculated field distribution of nonuniform Au nanoarray at different wavelength. (a) The simulation unit cell of nonuniform Au nanoarray. (b)-(d) the field distribution of nonuniform Au nanoarray.

Due the strong plasmon coupling in the adjacent nanowires with different length, the electric field tends to concentrate at some certain nanowire, which makes the maximum field enhancement of nouniform nanoarray is larger than that of uniform Au nanoarray (see Figure 3a and Figure S3). However, the nouniform field enhancement distribution may affect the stability and repeatability of Au nanoarray in the application of nanoantennas. Furthermore, the entire extinction intensity of Au nouniform nanoarray decreases dramatically (see Figure 2c) which may result in poor light absorption, so we believe the uniform nanoarray can be a better choice for nanoantennas which can be demonstrated by the PL and time-resolved PL measurements (see Figure 4, Figure S4, and Figure S5).

4. The avalanche multiphoton luminescence of Au nanoarray

The large field and LDOS enhancement can also be demonstrated by the photoluminescence (PL) measurement [4], so we compared the multiphoton luminescence of Au nanoarray grown by pulse AC and normal AC method. The samples are from the extinction samples in Figure 2a with nearly the same growth condition and the excitation wavelength was set to be 800 nm. Figure S4a is the PL spectra of Au nanoarray grown by pulse AC method, and we could easily see dramatically intensity enhancing as the excitation power increasing, which is caused by the avalanche multiphoton luminescence of Au nanoarray [5]. Figure 4b is the excitation power dependence of PL intensity. We can define the curve slope as v = ə log I_{PL} / log P_{exc}, and the critical excitation power of three photon luminescence

changing to avalanche multiphoton luminescence is P_c. It is obviously to see the Au nanoarray grown by pulse AC method displays larger slope v and lower P_c, which strongly indicates larger eclectic field and LDOS enhancement.

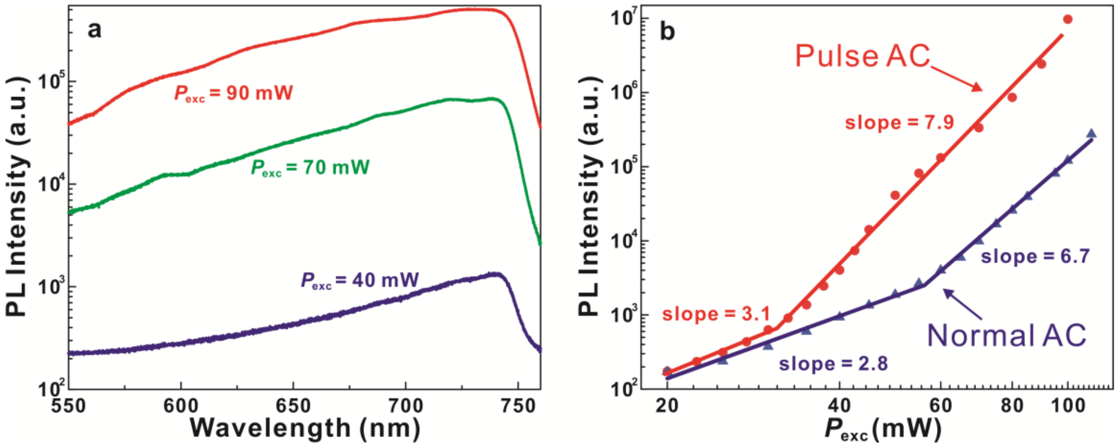

Figure S4. (a) PL spectra of Au nanoarray grown by pulse AC method. (b) The excitation power dependence of PL intensity from the Au nanoarray grown by pulse and normal AC method.

5. The emission rate manipulating of QDs with emission wavelength of 790 nm

Figure S5 displays the time-resolved PL spectra of QDs on the Au nanoarray and SiO_2 substrate. The emission wavelength of the QDs is 790 nm, and the excitation wavelength is 700 nm with a repetition rate of 7.9 MHz. From the Figure S5, The PL decay trace of the QDs on SiO_2 substrate is single exponential with the corresponding emission rate τ = 0.0138 ns^{-1} (lifetime being 72.3 nm). On the other side, the PL curves of the QDs on Au nanoarray decay in a two-component exponential form, which can be describe by the formula $I_{PL}(t) = A_f e^{-t/t_f} + A_s e^{-t/t_s}$, where A_f and A_s are the weight factors of the fast and slow decay processes, respectively; t_f and t_s are the corresponding lifetimes (emission rate $\tau = 1/t$).

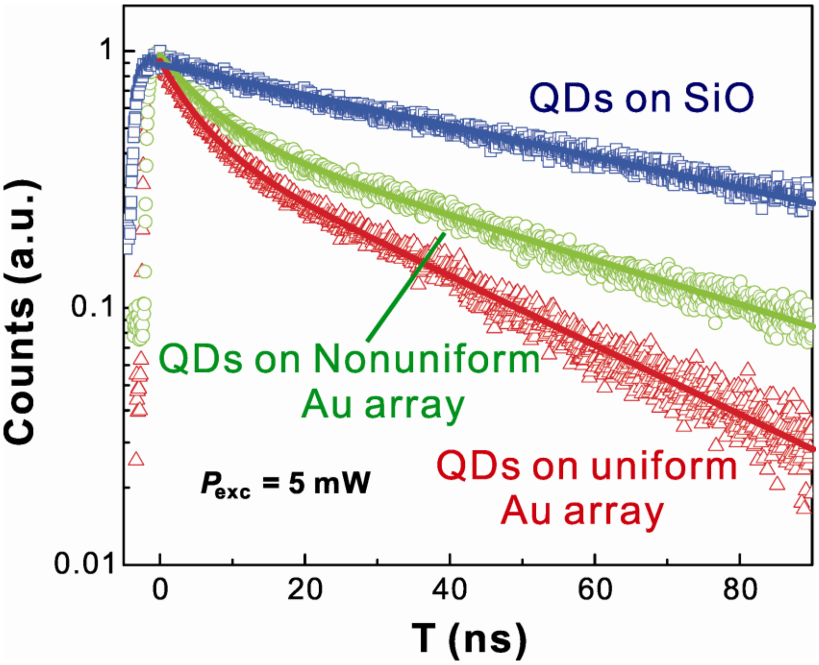

Figure S5. The normalized time-resolved PL spectra of QDs on Au nanoarray. The emission rate of QDs increase from 0.0138 ns^{-1} to 0.0833 ns^{-1} by the existence of Au nanoarray, showing an enhancement of 6.04 times.

We can calculate that the t_f is 11.7 ns and 12.0 ns, with the A_f rate (A_f /(A_f + A_s)) being 79.8% and 62.1% for the QDs on the Au nanoarray grown by pulse AC and normal AC, respectively. Thus the corresponding emission rate τ is 0.0854 and 0.0833 ns^{-1}. Considering the original decay rate of QDs is 0.0138, an enhancement of 6.2 times has been found in the nanosystem combined the QDs and uniform Au nanoarrays. We note that the average LDOS enhancement at 655 nm is smaller than that of QDs at 792 nm, but in our experiment the emission rate enhancement of QDs at 655 nm (QDs-655) is larger than that of QDs at 790 nm (QDs-790). This phenomenon may be caused by the assembling status of QDs on the AAO template loaded with Au nanoarray. Since the average diameter of QDs-790 is larger than of QDs-655, the distance between QDs-790 and Au nanoarray may be larger; on the

other hand, the LDOS enhancement is very sensitive to the distance (see Figure 3c). Therefore, the QDs-790 display smaller emission rate enhancement. The Au nanoarrays are the samples from Figure 2a. Since the results in Figure S5 are nearly the same with Figure 4, one can see the emission rate manipulating property of Au nanoarray is quite a common phenomenon, and this conclusion strongly suggests the application of Au nanoarray in the design of functional plasmonic nanoatenna.

Reference

[1] Payne, E.; Shuford, K.; Park, S.; Schatz, G.; Mirkin, C. *J. Phys. Chem. B* **2006**, 110, 2150-2154.

[2] Zhou, Z. K.; Li, M.; Yang, Z. J.; Peng, X. N.; Su, X. R.; Zhang, Z. S.; Li, J. B.; Kim, N. C.; Yu, X. F.; Zhou, L.; Hao, Z. H.; Wang, Q. Q. *ACS Nano* **2010**, 4, 5003–5010.

[3] Nielsch K.; Müller, F.; Li, A. P.; Gösele, U. *Adv. Mater.* **2000**, 12, 582–586.

[4] Zhou, Z. K.; Peng, X. N.; Yang, Z. J.; Zhang, Z. S.; Li, M.; Su, X. R.; Zhang, Q.; Shan, X. Y.; Wang, Q. Q.; Zhang, Z. Y. *Nano Lett.* **2011,** *11*, 49−55.

[5] Wang, Q. Q.; Han, J. B.; Guo, D. L.; Xiao, S.; Han, Y. B.; Gong, H. M.; Zou, X. W. *Nano Lett.* **2007**, 7, 723−728.
